# The genomic characterisation and comparison of *Bacillus cereus* strains isolated from indoor air

**DOI:** 10.1186/s13099-021-00399-4

**Published:** 2021-01-30

**Authors:** Balakrishnan N. V. Premkrishnan, Cassie E. Heinle, Akira Uchida, Rikky W. Purbojati, Kavita K. Kushwaha, Alexander Putra, Puramadathil Sasi Santhi, Benjamin W. Y. Khoo, Anthony Wong, Vineeth Kodengil Vettath, Daniela I. Drautz-Moses, Ana Carolina M. Junqueira, Stephan C. Schuster

**Affiliations:** 1 Singapore Centre for Environmental Life Sciences Engineering, Nanyang Technological University, Singapore, Singapore; 2 Departamento de Genética, Instituto de Biologia, Universidade Federal do Rio de Janeiro, Rio de Janeiro, Brazil

**Keywords:** *Bacillus cereus*, Food poisoning, Antibiotic resistance, Enterotoxins

## Abstract

**Background:**

*Bacillus cereus* is ubiquitous in nature, found in environments such as soil, plants, air, and part of the insect and human gut microbiome. The ability to produce endospores and biofilms contribute to their pathogenicity, classified in two types of food poisoning: diarrheal and emetic syndromes. Here we report gap-free, whole-genome sequences of two *B. cereus* strains isolated from air samples and analyse their emetic and diarrheal potential.

**Results:**

Genome assemblies of the *B. cereus* strains consist of one chromosome and seven plasmids each. The genome size of strain SGAir0260 is 6.30-Mb with 6590 predicted coding sequences (CDS) and strain SGAir0263 is 6.47-Mb with 6811 predicted CDS. Macrosynteny analysis showed 99% collinearity between the strains isolated from air and 90.2% with the reference genome. Comparative genomics with 57 complete *B. cereus* genomes suggests these strains from air are closely associated with strains isolated from foodborne illnesses outbreaks. Due to virulence potential of *B. cereus* and its reported involvement in nosocomial infections, antibiotic resistance analyses were performed and confirmed resistance to ampicillin and fosfomycin, with susceptibility to ciprofloxacin, tetracycline and vancomycin in both strains.

**Conclusion:**

Phylogenetic analysis combined with detection of haemolytic (*hblA, hblC,* and *hblD*) and non-haemolytic (*nheA, nheB*, and *nheC*) enterotoxin genes in both air-isolated strains point to the diarrheic potential of the air isolates, though not emetic. Characterization of these airborne strains and investigation of their potential disease-causing genes could facilitate identification of environmental sources of contamination leading to foodborne illnesses and nosocomial infections transported by air.

## Background

*Bacillus cereus* is a small rod-shaped, Gram-positive, facultatively anaerobic, motile, spore-forming bacteria [1] belonging to the *Bacillus cereus* sensu lato group [2] of the phylum Firmicutes. The “*Bacillus cereus* group” is comprised of at least eight closely-related species, but the most studied are *B. cereus, B. thuringiensis,* and *B. anthracis* due to their clinical and socio-economic importance [3]. Taxonomic classification of these organisms has long been controversial due their historical classification based on phenotypic traits [4] while DNA–DNA hybridization results shows that they hybridize beyond the 70% species defining limit [5] and share almost 99% similarity in their 16S ribosomal RNA (rRNA) gene sequences [6]. This close genetic similarity has led some authors to argue that the entire group should be considered as a single unique species with diverse strains that differs in plasmid content or gene expression [7].

*Bacillus cereus* was first isolated from air samples collected from a cowshed in 1887 [8] and has been reported in soil [9], plants [10], the intestine of insects [11] and animals [1], and also in clinical environments [12]. The ability to produce endospores and biofilms are crucial to allow *B. cereus* to endure heat and dehydration and hinder their removal from adhering surfaces [13, 14]. These characteristics also contribute to their pathogenicity, traditionally classified in two types of food poisoning: diarrheal and emetic syndromes [15]. More recently, *B. cereus* also has been identified as an etiological agent of localized wound and eye infections, as well as in systemic infections [3].

From 1998 to 2008, a total of 235 foodborne disease outbreaks (2050 illnesses in total) were reported to the Centers for Disease Control and Prevention (CDC) in the United States that were caused (881 cases) or suspected to be caused (1169 cases) by *B. cereus* [16]. More recently, the Annual Communicable Diseases Surveillance report published by the Ministry of Health (Communicable Diseases Surveillance report 2018) in Singapore documented 398 notifications of food poisoning involving 3165 cases in 2018 and *B. cereus* was among the top three suspects of the reported outbreaks. Several other investigations demonstrated that *B. cereus* contamination can arise after improper handling of food and raw vegetables, poor hygienic practices of food handlers, and also from polluted environments, including the air [17–19].

In this article we report the complete genomes of two *B. cereus* strains isolated from indoor air samples in Singapore, namely SGAir0260 and SGAir0263. A comparative analysis with 55 additional genomes of *B. cereus* and the identification of virulence genes provided a comprehensive analysis of the disease potential of SGAir strains and will be a valuable resource for investigations of foodborne and nosocomial infections potentially caused by strains transported by air in tropical environments, thus assisting the identification of sources and sinks of future outbreaks.

## Methods

### Isolation, DNA extraction and sequencing

*Bacillus cereus* strains SGAir0260 and SGAir0263 were isolated in 2015 from indoor air samples collected in a commercial building in Singapore. Using an Andersen single-stage impactor (SKC, USA), air was impacted onto Malt Extract Agar (Sigma-Aldrich, USA) and Potato Dextrose Agar (Sigma-Aldrich, USA) at 28.3 L/min for 3 min, and incubated at 30 °C for 3 days. Resulting colonies were sub-cultured on Tryptic Soy Agar (Sigma-Aldrich, USA) and the two strains were individually inoculated in lysogeny broth (LB, Becton–Dickinson, USA) at 30 °C overnight to obtain axenic cultures.

DNA was extracted using the Wizard genomic DNA purification kit (Promega, USA) following the manufacturer’s protocol and DNA quantitation was carried out with NanoDrop (Thermo Scientific) and QuantiFluor^®^ dsDNA system (Promega). Libraries were prepared based on the 20 kb SMRTbellTM Template Preparation Protocol (Pacific Biosciences) using 5 µg of purified, sheared DNA as input and assessed on an Agilent DNA 12,000 chip on a 2100 Bioanalyzer (Agilent) to determine the optimal cut-off for size selection. The library was then size-selected on a Sage Science Blue Pippin instrument, using a dye-free 0.75% agarose cassette and 15 kb as the cut-off and sequenced in one SMRTcell (SGAir0260) or two SMRTcells (SGAir0263) on a Pacific Biosciences RSII single-molecule real-time (SMRT) sequencing platform at a loading concentration of 0.2 nM.

Additionally, 300 bp paired-end reads were produced on the MiSeq platform (Illumina). Libraries preparation was performed according to TruSeq Nano DNA Sample Preparation protocol (Illumina). A total of 200 ng of genomic DNA was then sheared on a Covaris E220 (Covaris) to ~ 550 bp following the manufacturer’s recommendation, uniquely tagged with Illumina’s TruSeq HT DNA barcodes, and pooled for sequencing. The finished library was quantitated using QuantiFluor dsDNA assay (Promega) and the average library size was determined on an Agilent Tapestation 4200, followed by library dilution to 4 nM. The concentration of the diluted library was then validated by qPCR on a QuantStudio-3 real-time PCR system (Applied Biosystems), using the Kapa library quantification kit for Illumina platforms (Kapa Biosystems) prior to sequencing on the Illumina MiSeq platform at a read-length of 300 bp paired-end. Sequencing was perfromed at the Singapore Centre for Environmental Life Sciences Engineering (SCELSE) sequencing facility, located at Nanyang Technological University (http://www.scelse.sg/Page/sequencing-capacity).

### Genome assembly and annotation

Long reads were used for de novo genome assemblies performed after quality control using preAssembler filter v1 protocol, distributed with the Hierarchical Genome Assembly Process version 3 (HGAP3; Pacific Biosciences) [20] with default parameters. Short reads were trimmed using Cutadapt version 1.8.1 [21] with a Phred quality score threshold of q20 (parameters “-q 20–trim-n–minimum-length 30–match-read-wildcards”). Assemblies were polished using Quiver with default parameters [20] and draft assemblies were improved with short reads using Pilon version 1.16 using 300-bp MiSeq paired-end reads and following parameters (–tracks–changes–vcf–fix all–mindepth 0.1–mingap 10–minmq 30–minqual 20–K 47) [22]. The assemblies were tested for circularization using Circlator 1.1.4 [23]. Small contigs were verified as plasmids using BLASTn against NCBI’s nucleotide collection database and taxa identification was done using the Average Nucleotide Identity (ANI) method utilizing a custom PERL script [24]. Annotation of genomes was performed using Pathosystems Resource Integration Center (PATRIC) with the default annotation scheme [25]. All 55 complete genomes of *B. cereus* were downloaded (on August 14, 2019) from the NCBI RefSeq database and re-annotated through PATRIC for consistent comparison.

### Comparative genomics and phylogenetic analysis

Genes were assigned to PATRIC genus specific families (PLFam) and used to evaluate the core and pan genome. Clusters of Orthologous Groups (COG) annotation was performed to functionally classify the PATRIC annotated proteins, using NCBI’s Conserved Domain Database (CDD) [26] search tool (Web CD-Search). Genome visualization was done using Circos version 0.69-6 [27]. Collinearity between SGAir strains and the NCBI reference genome was analyzed in MCScanX with default parameters [28], using PATRIC annotated CDS as inputs.

The Maximum likelihood (ML) phylogenetic tree was reconstructed using both air-isolated strains and 55 *B. cereus* complete genomes. Codon Tree pipeline [29, 30] was used to align 1000 PATRIC’s PGFams single-copy genes (one max allowed deletion and one max allowed duplication). Individual protein and nucleotide sequences were aligned with MUSCLE [31] and Codon_align function of BioPython [32], respectively. PATRIC tree building statistics is in Additional file 1. The phylogenetic reconstruction was run in RAxML [33] with concatenated alignments using partitions extracted from PATRIC on a local cluster with 1000 rapid bootstrap replicates. Substitution models GTRCAT and WAGF were implemented for nucleotide and amino acid datasets after the best fitting model search was performed in PATRIC. The tree was annotated using the Interactive Tree of Life v 4.4.2 (iTOL) [34]. Isolate source information for each genome was obtained from NCBI’s isolate source field (part of PATRIC) and missing records were manually investigated at NCBI. These sources were classified and added to the tree as ‘Environmental’, ‘Clinical’, or ‘Other’.

### Antibiotic/antimicrobial resistance

Antibiotic resistance prediction was done using Resistance Gene Identifier [35] against the Comprehensive Antibiotic Resistance Database [35, 36] and ResFinder [37] using RGI’s ‘strict’ paradigm. Btyper [38] was used to cross-validate antimicrobial resistance findings and the results were confirmed using the minimum inhibitory concentration (MIC) for the following antibacterial agents: ampicillin, ciprofloxacin, fosfomycin, tetracycline and vancomycin. Tests for AMR were conducted using the broth dilution method [36] in 24-well flat bottom culture plates (performed in triplicate). Antibacterial agents were added at final concentrations of 1024 to 0.0156 µg/ml by serial dilution. The strains were cultured overnight in cation-adjusted Mueller–Hinton II Broth (MHB) (Becton–Dickinson, USA), and serially diluted and plated onto Muller Hinton II Agar (Becton–Dickinson, USA) to estimate the viable cell number as colony forming unit (CFU). The same culture was diluted in MHB using optical density at 600 nm (OD600) correlation to CFU to obtain final cell concentration of 5*105 CFU/ml. An equal volume of cells and antibacterial agent in MHB was added into each well. The plates were incubated at 35 °C for 20 h [39]. After incubation, OD600 was measured using a spectrophotometer. The lowest concentration of antibacterial agent with no detectable OD600 value (or growth) was recorded as the MIC and isolates were classified according to the Clinical and Laboratory Standards Institute (CLSI) guidelines (CLSI. 2016).

### Virulence factor prediction

Virulence factors were predicted using the Virulence Factor Database (VFDB) via the VFanalyzer pipeline accessed through the web application [40], uploading the chromosomal and plasmid sequences for each genome and using the NCBI reference strain ATCC 14579 as the representative genome. The virulence-based classification tool, Btyper, was also used to predict virulence genes and to perform multilocus sequence typing (MLST) and *rpoB* allelic typing.

### Quality assurance

To ensure axenic culture, a single colony was picked and repeatedly streaked on fresh TSA agar. The pure colony had the DNA extracted as previously described and sequenced on both PacBio RSII and Illumina MiSeq, using the Illumina short reads for polishing. Long reads were sequenced for strains SGAir0260 and SGAir0263 to a mean depth of 161× and 150×, respectively, and assembled after quality control using preAssembler filter v1 protocol, distributed with the Hierarchical Genome Assembly Process version 3, using default parameters. Sequenced short reads used for polishing were filtered using Phred quality score threshold of q20 and any resulting contigs containing lower than 10× coverage were investigated by BLASTn to NCBI nr/nt database and against the chromosomal genome to check for contamination or assembly issues (resulting read numbers are given in assembly statistics in Table 1). The 16S sequence was extracted from the completed assembly and screened for copies from multiple organisms. Taxa identification was performed using ANI and threshold of 95% confidence score.

**Table 1:** Genome assembly statistics and PATRIC annotations for *B. cereus* SGAir0260, SGAir0263, and NCBI reference strain ATCC 14579 (accession number GCF_000007825)

Statistics	SGAir0260	SGAir0263	ATCC 14579
Number contigs	8	8	2
Genome length (bp)	6,302,031	6,469,840	5,427,083
Genome GC content (%)	35	34.95	35.29
Chromosome length (bp)	5,945,556	5,947,158	5,411,809
PacBio RSII subreads	168,732	28,595	(N/A)
PacBio coverage (fold)	161.06	150.26	(N/A)
Illumina MiSeq PE reads	853,531	743,594	(N/A)
Illumina MiSeq Coverage (fold)	79.65	69.06	(N/A)
PATRIC rRNA (5S + 16S)	24	24	26
PATRIC CDS	6590	6811	5701
PATRIC tRNA	99	99	108
PATRIC repeat regions	182	249	74
PATRIC MLST Assignment	157	157	921

## Results and discussion

### Genome assembly and annotation

**Fig. 1:**
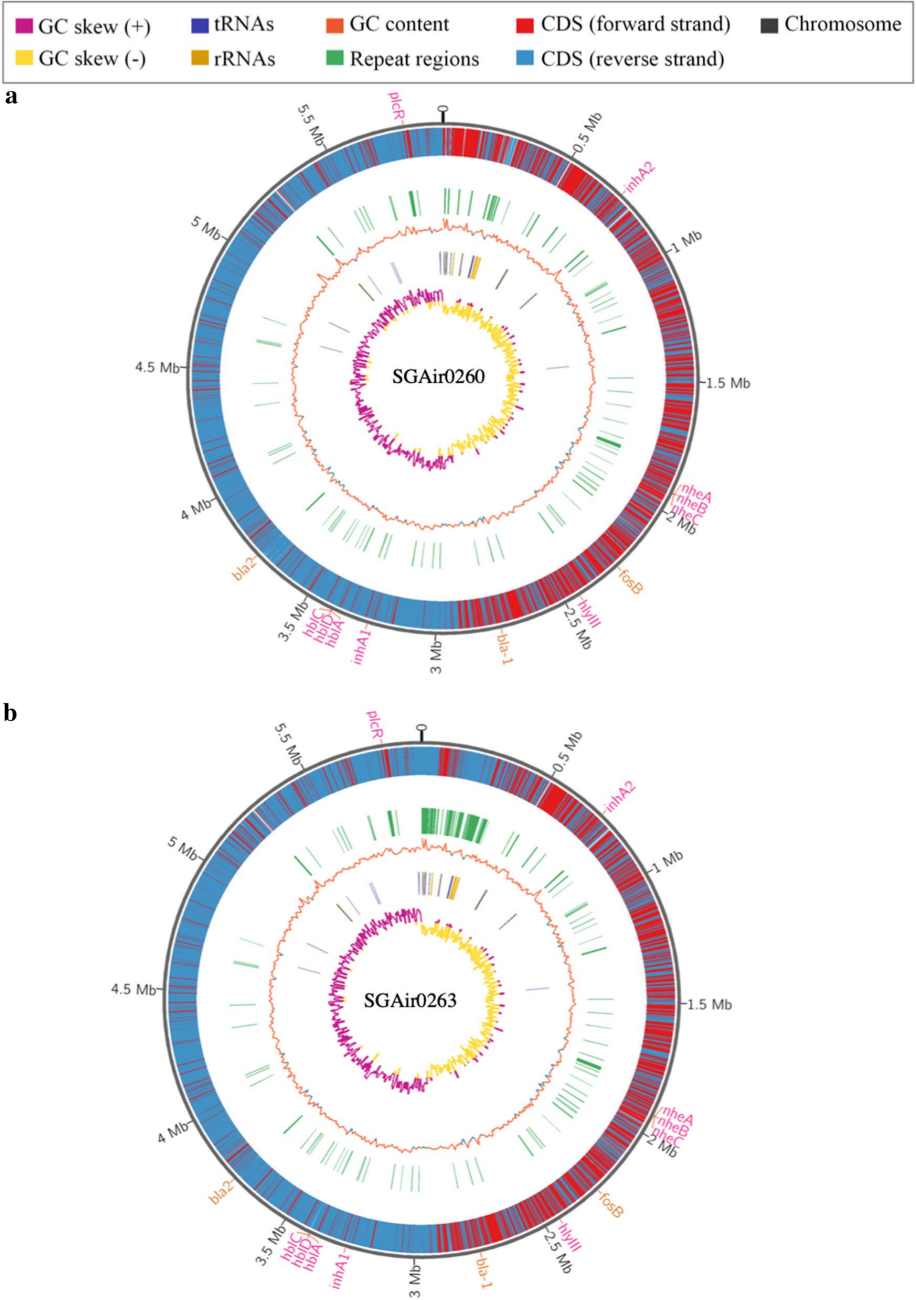
Circos plot showing PATRIC annotations for *Bacillus cereus* strains SGAir0260 (**a**) and SGAir0263 (**b**). The antibiotic resistance genes *bla1, bla2,* and *fosB* are displayed in light brown. The virulence genes *hblACD, nheABC, inhA,* and *plcR* are displayed in dark pink

The polished hybrid approach to assemble the genomes of *B. cereus* strains SGAir0260 and SGAir0263 allowed a supported PacBio genome coverage of 161.06 and 150.26-fold coverage, respectively. The strain SGAir0260 has one non-circularised chromosome and seven plasmids (three circular and four non-circular), totalling 6.3 Mb in size, while the strain SGAir0263 has one circularised chromosome and seven plasmids (two circular and five non-circular), totalling 6.5 Mb, compared to the 55 complete genome range of 5.2 Mb to 6.4 Mb with a mode of 2 contigs. Genome assembly statistics and PATRIC annotations are listed in Table 1 while graphical visualization of annotated genes, GC skew and GC content across the genomes are shown in Fig. 1. Graphical representation for plasmids are shown in Additional file 2. Taxonomic classification using ANI resulted in inference of species level identification to *B. cereu*s species with a high confidence score of 96.76% identity for SGAir0260 and 96.74% for SGAir0263 [41]. The strains isolated from air were clustered into the *B. cereus* clade with a robust node support (bootstrap = 100), as seen in the Additional file 3.

### Comparative genomics and phylogenetic analysis

Gene family comparison using PATRIC’s genome annotation of the 55 complete *B. cereus* genomes from NCBI revealed 3330 core PLFam protein family groups and 18,061 PLFams participating in the pan-genome of the combined 57 genomes (see Additional file 4). There were 13 strain-specific PLFams for SGAir0260 comprising 13 proteins, while strain SGAir0263 presented 31 strain-specific PLFams related to 31 proteins with largely hypothetical protein annotation (see Additional file 5). Functional classification of genes into COG categories is displayed in Additional file 6. The most number of genes were assigned to Transcription (category K) and Amino acid transport and metabolism (category E).

Pairwise macrosynteny analysis between the NCBI reference strain ATCC 14579 and each SGAir strain using the PATRIC annotated CDS (Table 1) show syntenic blocks comprising of approximately 4948 CDS gene pairs with SGAir0260 and 4947 CDS gene pairs with SGAir0263 (corresponding to approximately 88.77% and 88.75% of the reference genome respectively). The resulting dot plot showing the mapped regions of SGAir strains against the reference genome is given in Additional file 7 (Figure I.a and II.a) and further visualization displays a region of rearrangement in the chromosome of both the SGAir strains compared to the reference genome (Additional file 7 (Figure I.b and II.b). Investigation of this region shows that it is composed mostly of phage proteins (60.7%) and hypothetical proteins (19.1%) (Additional file 8). Differences between the reference and SGAir strains can be partially attributed to the large genome size differences, most notably the difference in non-chromosomal contigs, resulting in non-syntenic regions containing 409 CDS and 609 CDS in each SGAir strain, compared to that of 26 CDS for reference strain. A list of genes not found in the macrosyntenic analysis as gene pairs can be found in Additional file 8.

**Fig. 2:**
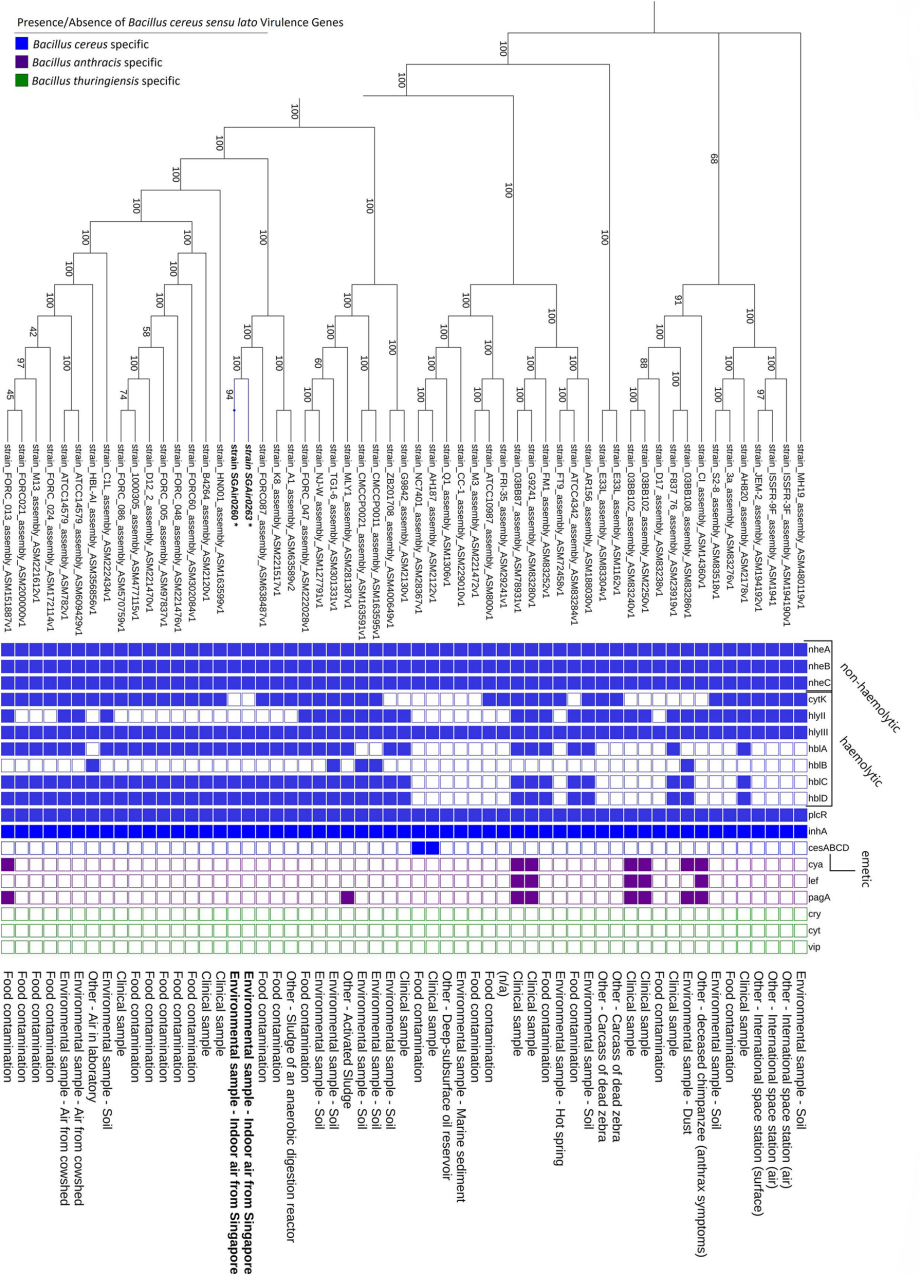
Maximum likelihood unrooted tree of SGAir0260 and SGAir0263 with 55 complete genomes retrieved from NCBI. The grid indicates the presence (filled square) and absence (clear square) of virulence genes specific *to B. cereus* group. Virulence genes related to *B. cereus* (blue), *Bacillus anthracis* (purple), and *Bacillus thuringiensis* (green) were analysed and are represented in the grid. The source of each isolate according to PATRIC and NCBI records and sequenced strain are shown on the right of the grid. Bootstrap supports (1000 replicates) are indicated above nodes

The ML phylogenetic tree generated shows that both strains group together with robust support (bootstrap = 94) and are closely-related to the strains FORC087, A1, and K8 (Fig. 2). These three isolates were isolated from “food contamination” outbreaks (chives for FORC087 and fermented Korean food for K8) and “activated sludge” for strain A1 (Fig. 2). Interestingly, strains SGAir0260 and SGAir0263 are more related to other *B. cereus* strains associated with foodborne illnesses than with those isolated from environmental samples such as soil and air. Subsequent *rpoB* allelic type assignment, originally designed to help track the source of food spoilage microorganisms in the milk supply chain [42], showed both strains as AT0154, the same type as predicted for neighbouring strains A1 and FORC087, though differing from strain K8 as AT0424. While two strains of the closely-related group share the same *rpoB* allelic type, the results of in silico MLST, based on multiple house-keeping genes (including *rpoB*), assign SGAir0260 and SGAir0263 strains to type ST157, while strains A1, FORC087, and K8 are assigned to type ST1001, ST446, and ST138 respectively. The complete list and accession numbers of genomes retrieved from NCBI are in Additional file 9.

### Antibiotic/antimicrobial resistance

*Bacillus cereus* is amongst other multi-drug resistant microorganisms whose antibiotic resistance mechanism varies between strains. They are generally resistant to penicillin and cephalosporins due to beta-lactamase production [15, 43]. Results from RGI gave hits to three antimicrobial resistance ontology (ARO) groups: (i) *fos*B gene (99.28% identity), related to inactivation of the antibiotic fosfomycin; *bc*I (95.5% identity), and (iii) *bc*II (90.59% identity), both related to beta-lactamase genes. Search for acquired antibiotic resistance using ResFinder found a match to fosfomycin resistance gene, *fos*B1 (99.04% identity) in both *B. cereus* SGAir strains. The AMR prediction by BTyper corroborated these predictions, finding *fosB1* with 99.04% identity. Additionally, BTyper also indicated the presence of *bla* genes, *bla1* (91.68% identity) and *bla2* (90.83% identity), which confer resistance to beta-lactams in both SGAir strains. The gene coverage was more than 97% for all predictions and results with less than 90% identities were not taken into consideration. Comparative analysis of the antibiotic resistance genes found in *B. cereus* SGAir strains shows a similar pattern reported for 16 of 55 complete *B. cereus* genomes, containing the *fosB*, *bla1*, and *bla2* at the above threshold (data not shown).

The antibiotic resistance predicted for the two *B. cereus* SGAir strains was further validated with experimental data establishing the MIC. Results confirmed resistance to ampicillin and fosfomycin, with susceptibility to ciprofloxacin, tetracycline and vancomycin (see Additional file 10). Taken together, the results from genome analysis and MIC experiments suggest that *B. cereus* strains SGAir0260 and SGAir0263 are resistant to ampicillin and fosfomycin due to its capability of producing beta-lactamase and the presence of *fos*B cassette.

### Virulence factors

Virulence genes associated with *B. thuringiensis*, (insecticidal genes *cry, cyt*, and *vip*), and *B. anthracis* (anthrax genes *cya, lef, pagA*) were absent in both *B. cereus* SGAir strains [44]. Though, the following toxins associated with diarrheal syndrome were found in the *B. cereus* SGAir strains: the haemolytic enterotoxins hemolysin BL (*hblACD*) and hemolysin III (*hyl III*), and the non-haemolytic enterotoxin locus *nheABC.* Despite the size and extra-chromosomal contig number of the *B. cereus* SGAir strains, none of the seven emesis linked cereulide synthetase (*cesHPTABCD*) genes were detected in our analysis [45, 46] for either strain.

In addition, the immune inhibitor A metalloprotease (*inhA*) was detected. This gene may help *B. cereus* survive in harsh, nutrient-poor environments by enabling the bacteria to escape macrophages after ingestion [47]. Both *B. cereus* SGAir strains had the pleiotropic regulator gene (*plcR*) which is known to participate in the regulation of many virulence [48–50] and quorum sensing genes [51]. The regulator *plcR* and paralogues were described previously for *B. cereus* ATCC 14579 and could be involved in the regulation of hundreds of genes [52]. While *plcR* is not responsible for all of *B. cereus*’ potential virulence, it is thought to allow it to respond and adapt to changing host environments [47, 51]. The presence of these virulence genes, as predicted from VFAnalyzer, can also be seen in Figs. 1 and 2.

Although the presence of these genes does not provide a direct indication of the strains’ actual pathogenicity [53], we suspect that these two strains have diarrheic potential but not emetic potential. Further cytotoxic activity assays will need to be performed to verify the virulence of both strains.

## Conclusion

While the members of the *B. cereus* species have been found to be ubiquitous in nature, the rise of the organism’s implication in nosocomial infections and known contamination of foodstuffs increases the advantages of characterizing environmentally obtained *Bacillus* isolates. This understanding could lend itself to help differentiate possible modes of infection such as indirect (air) or direct (touch or surface) and more precisely delineate between pathogenic and non-pathogenic strains. The current placement of these two isolates in relation to other complete genomes show them as being more closely grouped with other *B. cereus* strains isolated from cases of foodborne illness. The additional detection of haemolytic (*hblA*, *hblC*, and *hblD*) and non-haemolytic (*nheA*, n*heB*, and *nheC*) enterotoxin genes in both air-isolated strains point to the diarrheic potential of the air isolates, though possibly not emetic (due to the lack of *cytK* and *ces* genes). This study’s further evaluation of synteny and functional gene classification leverage the advantages of whole genome sequencing and characterization, allowing the creation of a more complete reference for future comparisons.

## Supplementary information

**Additional file 1.** Table of PATRIC Codon Tree Statistics. PATRIC’s codon tree analysis statistics for SGAir0260 and SGAir0263 strains.

**Additional file 2.** Circos plot showing plasmid sequences. Circos plot showing plasmid sequences of strains SGAir0260 and SGAir0263.

**Additional file 3.** Phylogenetic tree of 57 genomes with outgroups. Maximum likelihood tree of SGAir strains, 55 complete genomes of *Bacillus cereus* retrieved from NCBI, with outgroups, *Escherichia coli* 157 H7 str. Sakai, *Bacillus subtilis* subsp. subtilis str., and *Bacillus subtilis* subsp. spizizenii TU-B-10.

**Additional file 4.** Table of PATRIC PLFams participating in pan-genome and core-genome. List of PLFams revealed during PATRIC’s gene family comparison with 57 genomes, including the SGAir strains under study.

**Additional file 5.** Table of PATRIC PLFams specific to SGAir0260 & SGAir0263 strains. List of strain specific (SGAir0260 and SGAir0263) PLFams detected in PATRIC analysis.

**Additional file 6.** Functional annotation of SGAir strains (COG categories). Bar chart showing COG categories and number of conserved domains found for each category in the genomes of *Bacillus cereus* SGAir strains.

**Additional file 7.** Macrosynteny analysis results between NCBI reference strain and SGAir strains. Images showing the collinearity between the reference genome ATCC 14579 and strains SGAir0260 and SGAir0263, respectively.

**Additional file 8.** Table(s) of Non-gene pairs, specific to NCBI Reference Strain ATCC 14579 and SGAir strains, SGAir0260 and SGAir0263. The list of CDS not found in macrosyntenic analysis gene pairs for strains ATCC 14579, SGAir0260, and SGAir0263, including location along each contig. Includes annotation of genes found in strain ATCC 14579 for region involved in rearrangement.

**Additional file 9.** Table of 55 NCBI Complete Genome Assembly Stats and PATRIC Annotation. The complete list of genomes downloaded from NCBI and their accession numbers with PATRIC annotations.

**Additional file 10.** MIC for antibacterial agents in accordance to CLSI Standard Guideline M45A2E & M45-P. MIC experiment results for antibacterial agents in accordance to CLSI Standard Guideline M45A2E & M45-P.

## Data Availability

This project has been deposited at the NCBI GenBank under the BioProject accession PRJNA388547 with BioSample accession SAMN08222711 for SGAir0260 and BioSample accession SAMN08222727 for SGAir0263.

## Abbreviations

ANI: Average nucleotide identity
ARO: Antimicrobial resistance ontology
CARD: Comprehensive Antibiotic Resistance Database
CDC: Centers for Disease Control and Prevention
CDD: NCBI’s Conserved Domain Database
CLSI: Clinical and Laboratory Standards Institute
COG: Clusters of Orthologous groups
CRF: Colony forming unit
HGAP3: Hierarchical Genome Assembly Process version 3
iTOL: Interactive Tree of Life v 4.4.2
LB: Lysogeny broth
MHB: Mueller-Hinton II Broth
MIC: Minimum inhibitory concentration
MLST: Multilocus sequence typing
OD600: Optical density at 600 nm
PATRIC: Pathosystems Resource Integration Center
PLFam: PATRIC genus specific families
SMRT: Single-molecule real-time
VFDB: Virulence Factor Database
