# Supplementary material for: The genomic characterisation and comparison of Bacillus cereus strains isolated from indoor air
Source: Gut Pathog. 2021 Jan 30;13:6. doi: 10.1186/s13099-021-00399-4 (PMC7847026; doi:10.1186/s13099-021-00399-4)
Supplement: Supplementary file 2 — Additional file 2. Circos plot showing plasmid sequences. Circos plot showing plasmid sequences of strains SGAir0260 and SGAir0263. [file 13099_2021_399_MOESM2_ESM.pdf]

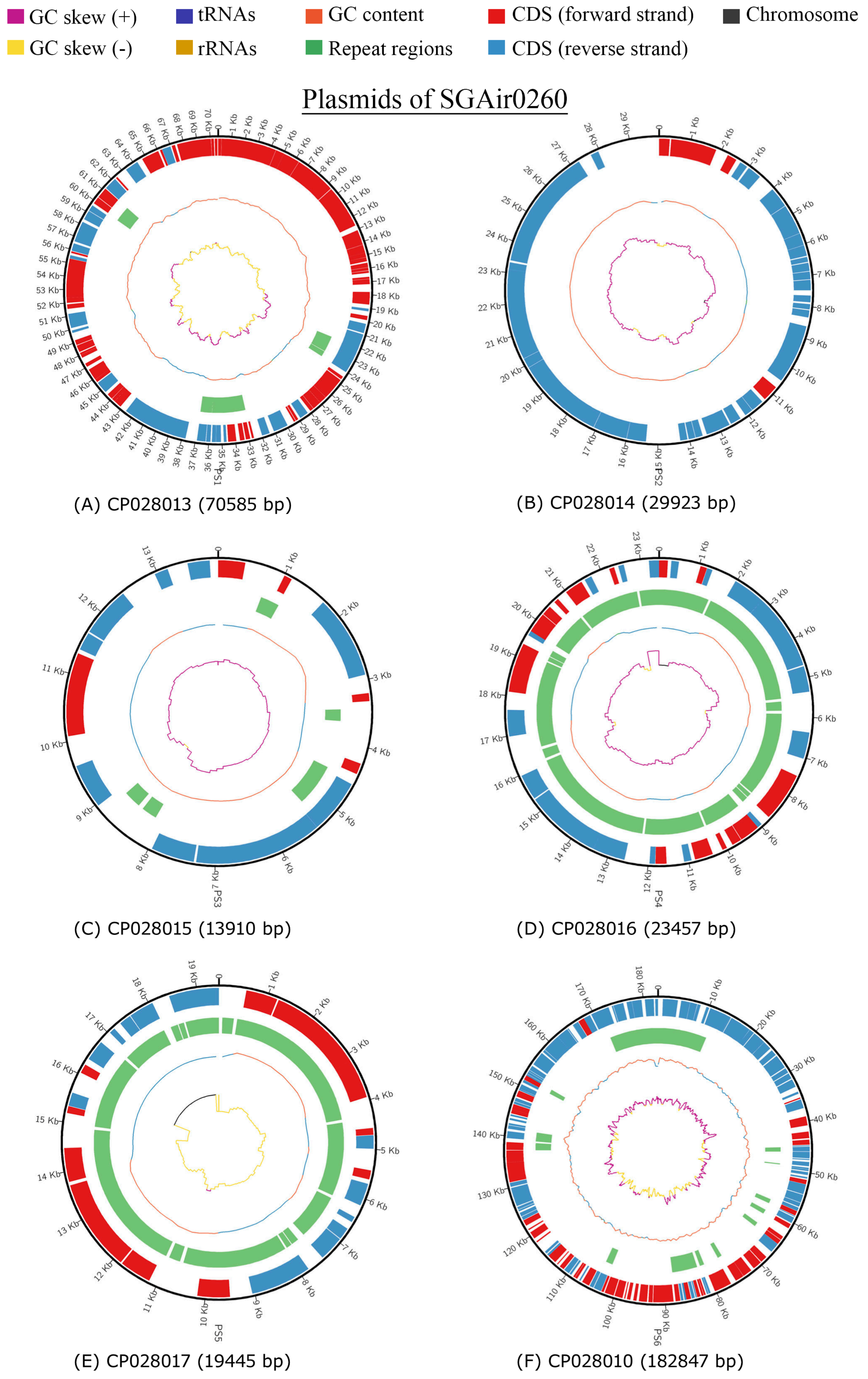
GC skew (+)
GC skew (-)
tRNAs
rRNAs
GC content
Repeat regions
CDS (forward strand)
CDS (reverse strand)
Chromosome
Plasmids of SGAir0260
PS1
(A) CP028013 (70585 bp)
PS2
(B) CP028014 (29923 bp)
PS3
(C) CP028015 (13910 bp)
PS4
(D) CP028016 (23457 bp)
PS5
(E) CP028017 (19445 bp)
PS6
(F) CP028010 (182847 bp)

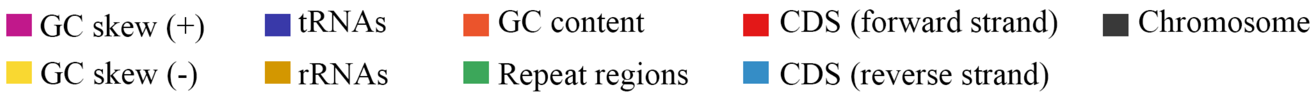

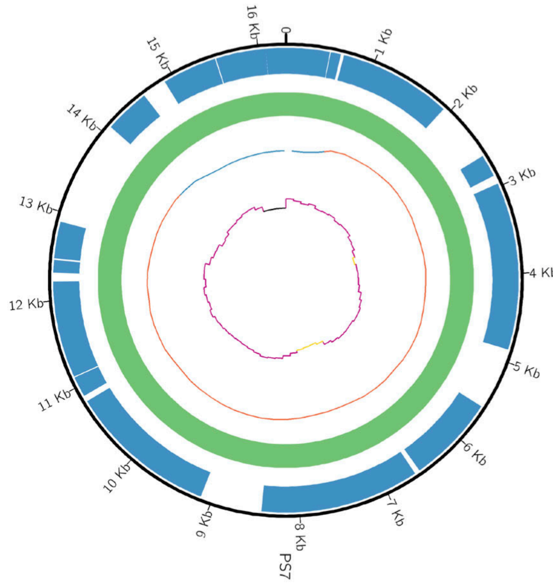

(G) CP028011 (16308 bp)

Plasmids of SGAir0263

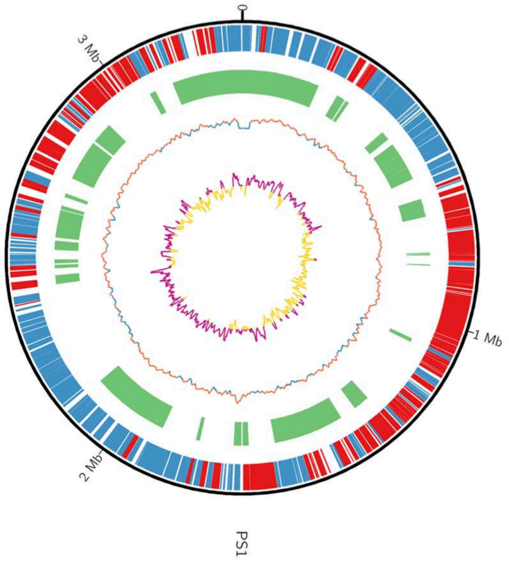

(H) CP027921 (333192 bp)

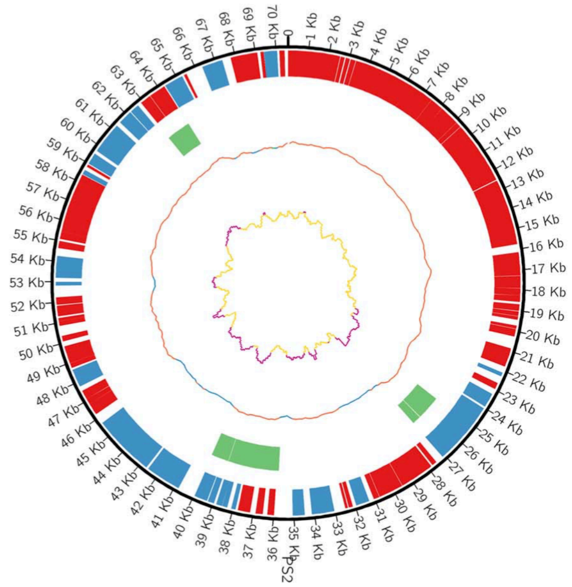

(I) CP027922 (70583 bp)

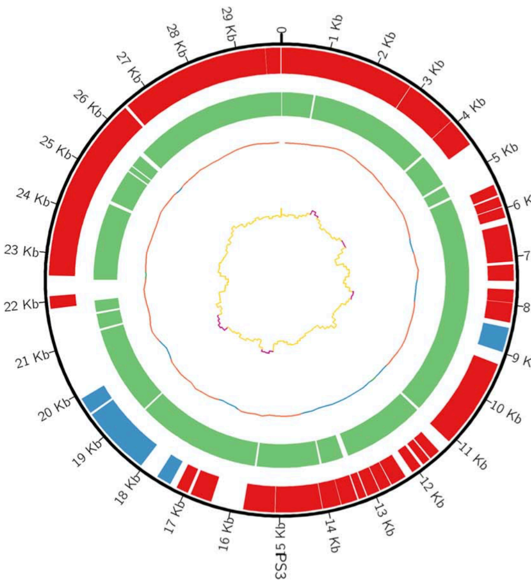

(J) CP027923 (29922 bp)

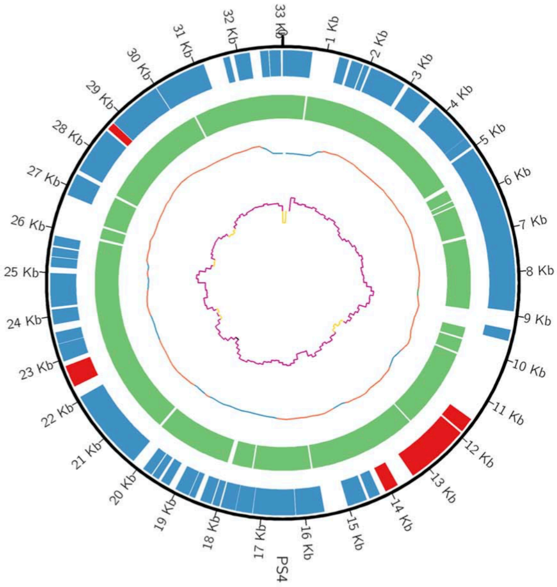

(K) CP027924 (33018 bp)

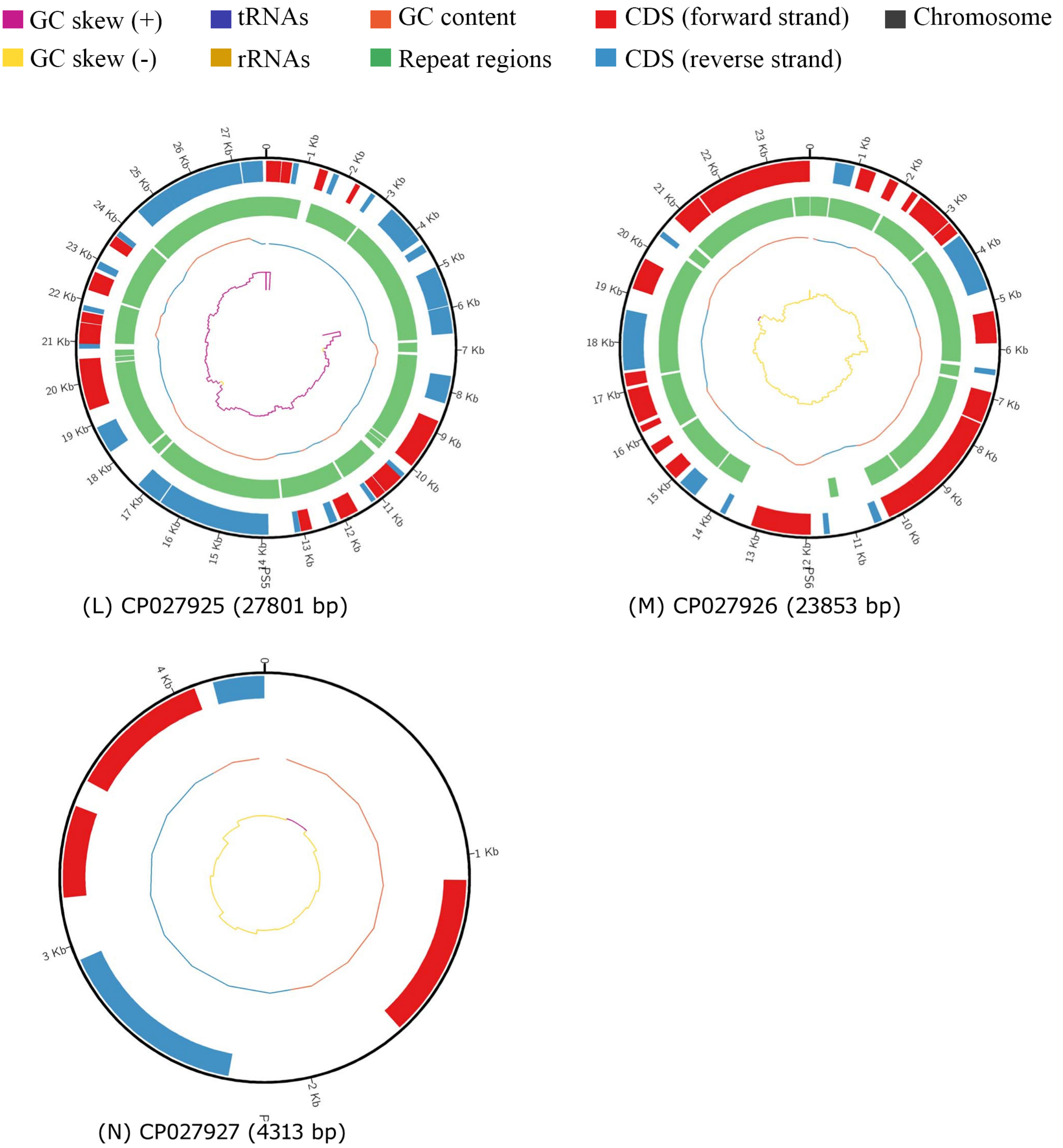

Additional File 2. Circos plot showing plasmid sequences of strains SGAir0260 (A to G) strain followed by SGAir0263 (H to N)). Circles from inside out: GC skew (magenta colour shows positive skew and bright yellow shows negative skew), tRNAs (dark violet) and rRNAs (dark yellow), GC content (orange ring), repeat regions (green bands), CDS on the forward (red) and reverse (dark blue) strand, chromosome band (black ring), genome size in Mb.
