# Supplementary material for: The genomic characterisation and comparison of Bacillus cereus strains isolated from indoor air
Source: Gut Pathog. 2021 Jan 30;13:6. doi: 10.1186/s13099-021-00399-4 (PMC7847026; doi:10.1186/s13099-021-00399-4)
Supplement: Supplementary file 3 — Additional file 3. Phylogenetic tree of 57 genomes with outgroups. Maximum likelihood tree of SGAir strains, 55 complete genomes of Bacillus cereus retrieved from NCBI, with outgroups, Escherichia coli 157 H7 str. Sakai, Bacillus subtilis subsp. subtilis str., and Bacillus subtilis subsp. spizizenii TU-B-10. [file 13099_2021_399_MOESM3_ESM.pdf]

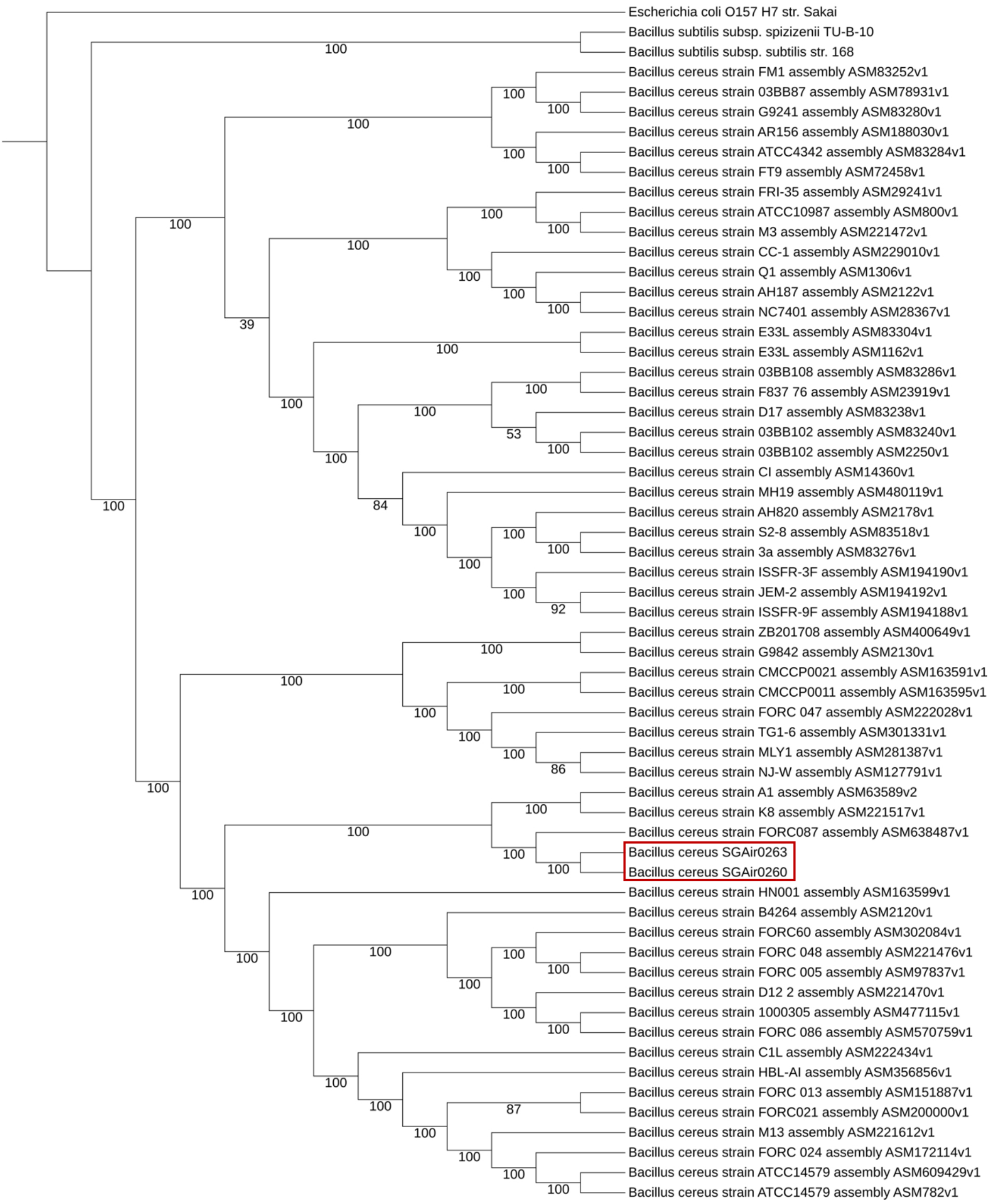

Additional File 3. Maximum likelihood tree of SGAir strains, 55 complete genomes of Bacillus cereus retrieved from NCBI, with outgroups, Escherichia coli 157 H7 str. Sakai, Bacillus subtilis subsp. subtilis str., and Bacillus subtilis subsp. spizizenii TU-B-10.
