# Supplementary material for: The genomic characterisation and comparison of Bacillus cereus strains isolated from indoor air
Source: Gut Pathog. 2021 Jan 30;13:6. doi: 10.1186/s13099-021-00399-4 (PMC7847026; doi:10.1186/s13099-021-00399-4)
Supplement: Supplementary file 6 — Additional file 6. Functional annotation of SGAir strains (COG categories). Bar chart showing COG categories and number of conserved domains found for each category in the genomes of Bacillus cereus SGAir strains. [file 13099_2021_399_MOESM6_ESM.pdf]

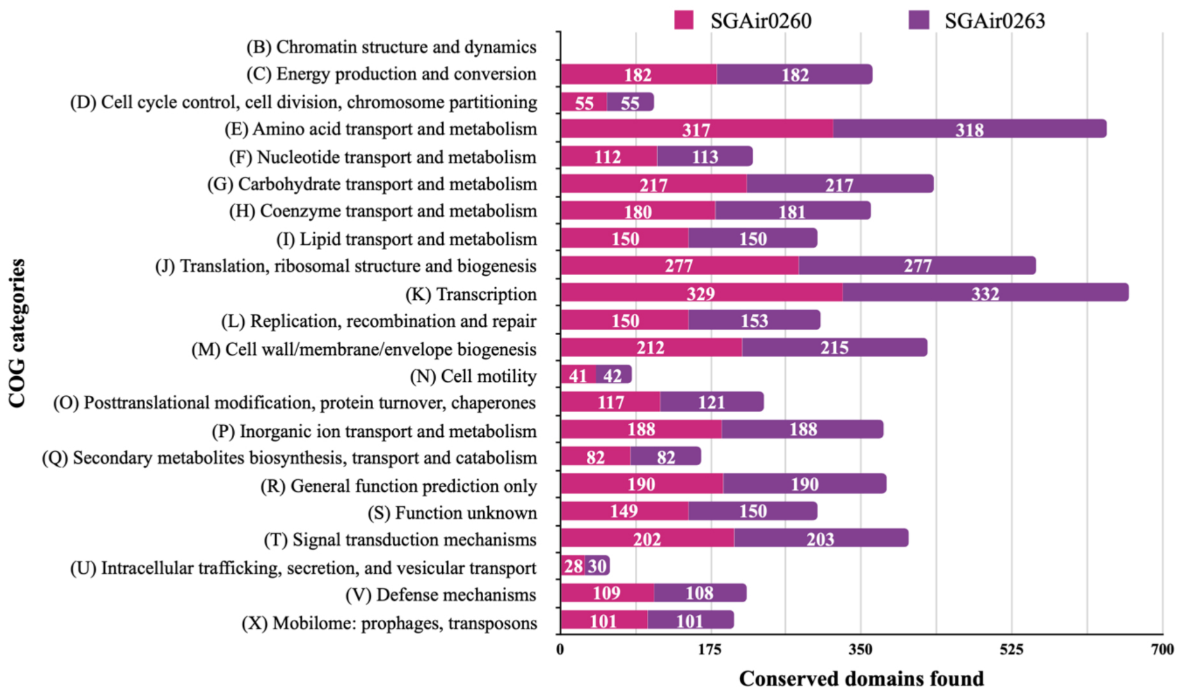

Additional File 6. Horizontal bar chart showing COG categories and number of conserved domains found for each category in the genomes of Bacillus cereus strains SGAir0260 (total number of genes assigned to COG groups = 3389) and SGAir0263 (3409). The number of identified conserved domains is shown inside each bar.
