# Supplementary material for: The genomic characterisation and comparison of Bacillus cereus strains isolated from indoor air
Source: Gut Pathog. 2021 Jan 30;13:6. doi: 10.1186/s13099-021-00399-4 (PMC7847026; doi:10.1186/s13099-021-00399-4)
Supplement: Supplementary file 7 — Additional file 7. Macrosynteny analysis results between NCBI reference strain and SGAir strains. Images showing the collinearity between the reference genome ATCC 14579 and strains SGAir0260 and SGAir0263, respectively. [file 13099_2021_399_MOESM7_ESM.pdf]

**I.a)** *B.c.*SGAir0260 vs *B.c.* ATCC 14579

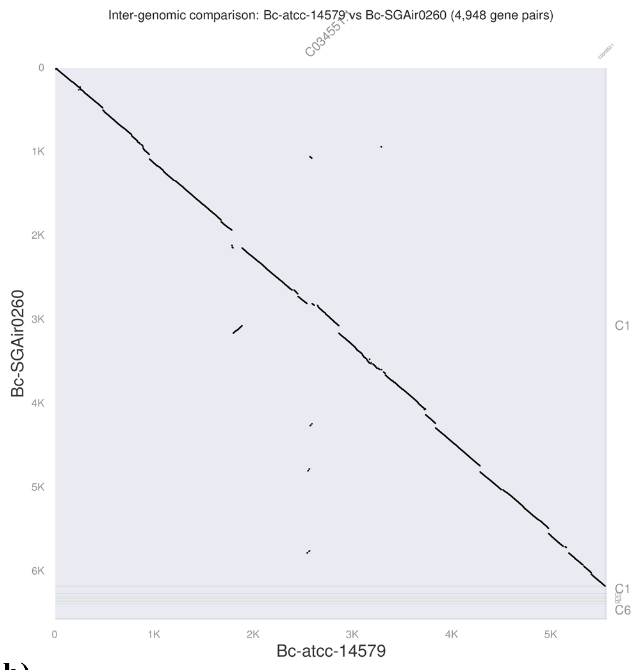


**II.a)** *B.c.*SGAir0263 vs *B.c.* ATCC 14579

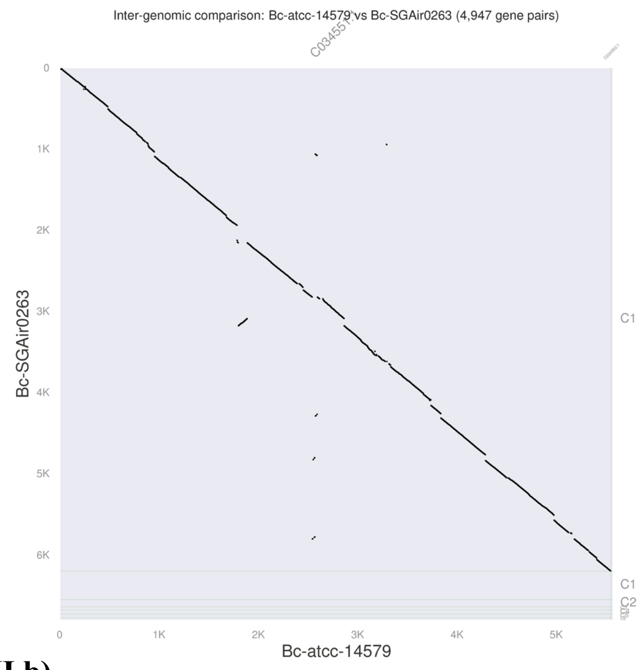


**I.b)**

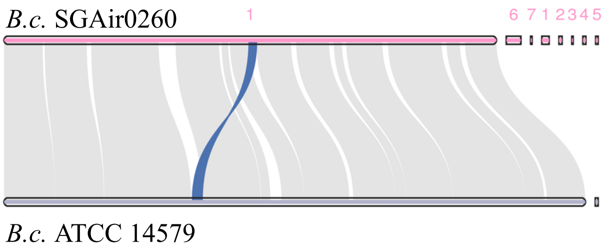


**II.b)**

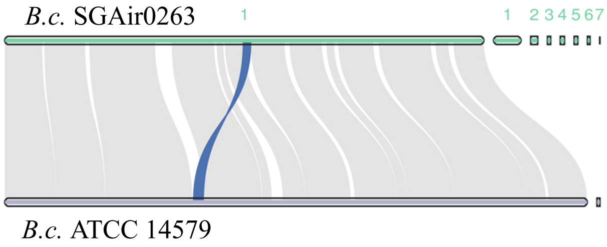


**III.a)** *B.c.*SGAir0260 vs *B.c.* SGAir0263

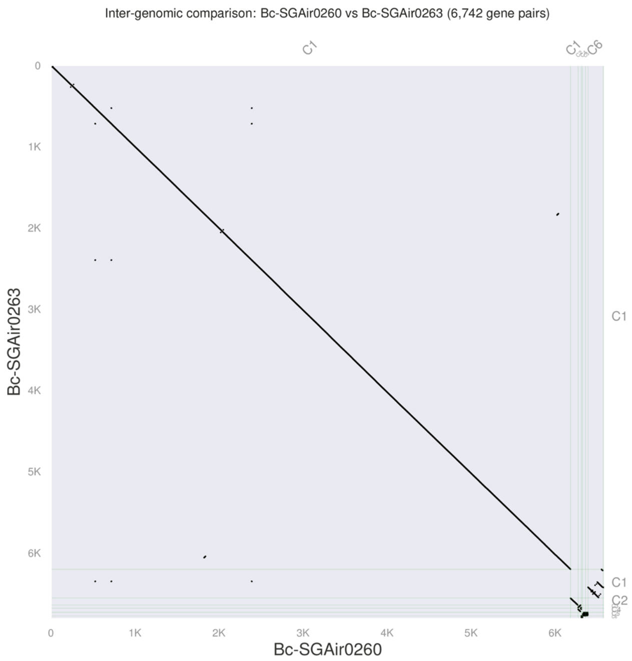


**IV)** SGAir strains vs *B.c.* ATCC 14579

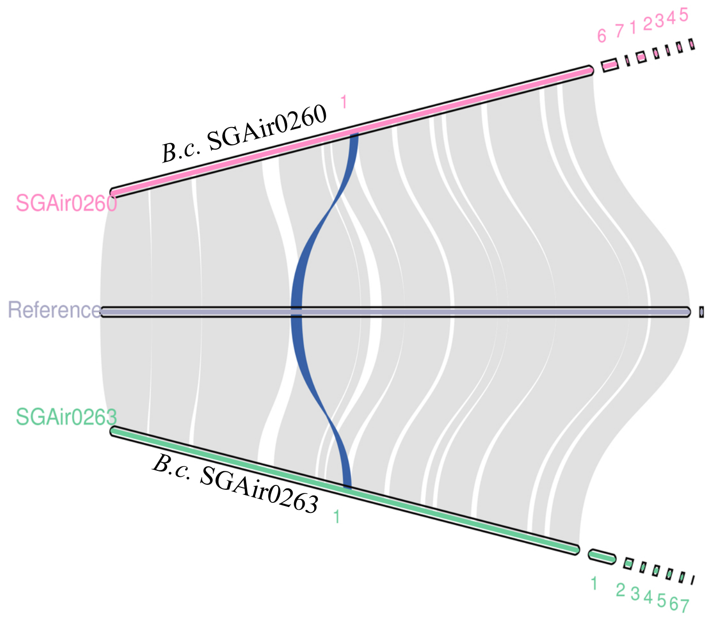


**III.b)**

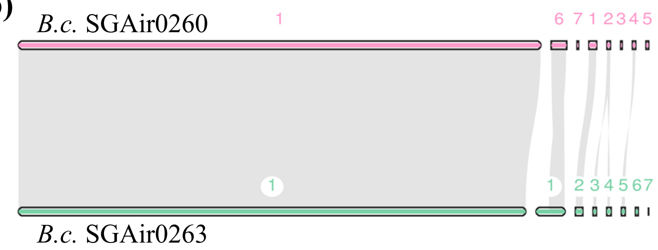

**Additional File 7**. Images I and II show the collinearity between the reference genome ATCC 14579 and strains SGAir0260 and SGAir0263, respectively. Each dot or line in the dot plot represents a syntenic region between the genomes. The diagonal lines in the dot plot (I.a and II.a) shows that their sequences are matching in these regions of the genome (stringent cut-off value used = 0.99). Small breaks along the diagonal lines indicate parts of the genome which are missing between the sequences. The same results are depicted using a linear line graph (I.b and II.b) and blue line representing the region not in order with the reference. Image III represents the collinearity between SGAir0260 and SGAir0263 strains. Image IV is a merged version of I.b and II.b. Note that genome sizes are scaled relative to each other and are not representative of their true size differences.
